# Treatment of Ruptured Giant Omphalocele and Gastroschisis with Liver Herniation using a Wound Retractor as a Novel Approach

**DOI:** 10.1055/s-0040-1721054

**Published:** 2020-12-15

**Authors:** Jana Nelson, Robin Wachowiak, Manuela Siekmeyer, Matthias Knuepfer, Ulrich Thome, Stepan Holger, Martin Lacher

**Affiliations:** 1 Division of Pediatric Surgery, Leipzig University Hospital for Children and Adolescents, Leipzig, Saxony, Germany; 2 Department of Pediatric Medicine, Leipzig University Hospital for Children and Adolescents, Leipzig, Saxony, Germany; 3 Division of Obstetrics and Gynaecology, University Hospital Leipzig Department of Obstetrics and Gynaecology, Leipzig, Saxony, Germany

**Keywords:** ruptured giant omphalocele, giant gastroschisis, liver herniation, abdominal wall defects, staged surgical repair

## Abstract

Ruptured giant omphaloceles (GO) and gastroschisis with total liver herniation are rare cases of exceptionally large abdominal wall defects. Many of these children have lethal outcome. The surgical and postsurgical management are complex. We report on two cases treated with staged surgical repair using a wound retractor as a silo. With this technique, the liver and intestines could be reduced into the abdomen with secondary closure of the abdominal cavity within the first 1 to 2 weeks of life.

## Introduction

The incidence of giant omphalocele (GO) is very low. An exact number is unknown as there is no consensus on the definition of GO. Many authors define GO as an abdominal wall defect larger than 5 cm in diameter and herniation of >50% of the liver (hepatoomphalocele), 1 others as an abdominal wall defect, in which a primary closure is not possible. 2 Due to associated comorbidities in children with omphaloceles such as pulmonary hypertension, pulmonary hypoplasia, and cardiac malformation, the mortality rate remains as high as 17 to 41%. 3 The best initial management of GO is still controversial. If primary repair is not possible, surgical options include staged repair or conservative management with delayed closure. 2 4 5 In 7 to 23% of all fetuses, rupture of an omphalocele occurs—which may happen antenatally, during delivery or postnatally; the risk of rupture is greater in GO. 6 If an omphalocele sac is ruptured, conservative management is no longer an option.

The term “giant gastroschisis” is used in for gastroschisis with complete liver herniation. Giant gastroschisis is extremely rare (6% of patients with gastroschisis) with usually lethal outcome due to pulmonary hypoplasia, which may result from an in utero deformation sequence. 7 8 Due to the displacement of the liver, the molding of the lower thoracic cage is not properly induced. Decreased intra-abdominal pressure also causes a paradoxical breathing movement with collapse of lower rib cage inward and inability to develop negative intrathoracic pressure during inspiration.

In both ruptured GO and gastroschisis with liver herniation, surgical and postsurgical management is complex. We report on two cases in which we applied a novel technique using a wound retractor as a silo.

## Case 1

**Fig. 1:**
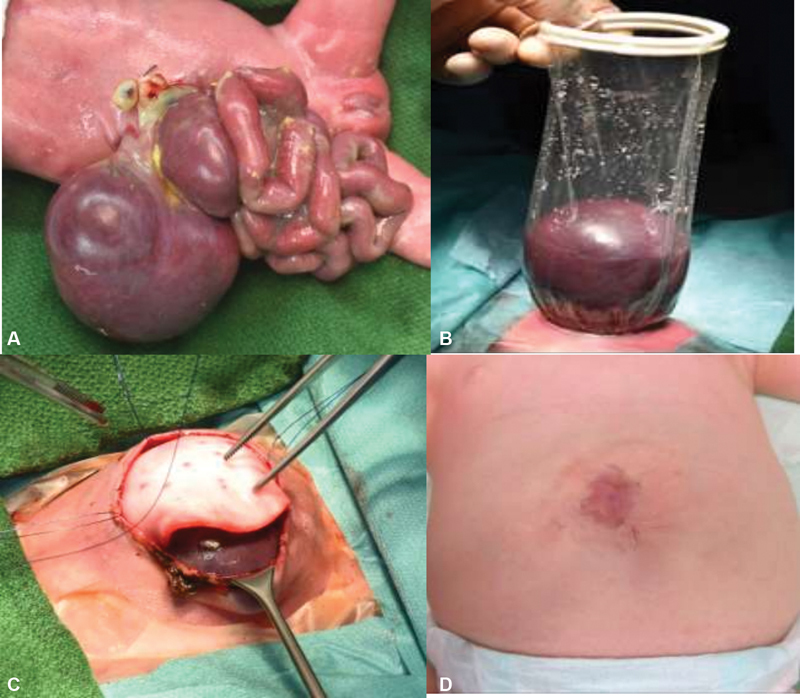
Giant gastroschisis ( **A** ) after delivery, ( **B** ) after positioning of the wound retractor under the fascia, ( **C** ) secondary closure of the abdominal cavity using a bovine xenograft on day of life 10, and ( **D** ) follow-up at the age of 14 months.

A female infant (gestational age of 33 weeks and birthweight of 1,630 g) was born via C-section due to premature rupture of membranes with prenatally diagnosed hepato-omphalocele. At birth, a fascial defect to the right of the umbilical cord (gastroschisis) with prolapse of intestine, stomach, spleen, and liver was seen ( Fig. 1A ). After delivery, a wound retractor (Alexis wound retractor, size XS, Applied Medical) was positioned under the fascia as a silo and the intestines reduced into the abdomen ( Fig. 1B ). In the neonatal intensive care unit, we were able to reposition the liver into the abdominal cavity by daily reduction under mechanical ventilation with mild pressures and sedation. On day of life (DOL) 10, we performed a secondary closure of the fascial defect (6 cm) using a xenograft (bovine dermis, SurgiMed collagen matrix, 2 mm thickness; Fig. 1C ) and applied a vacuum assisted closure-dressing for 10 days. Postsurgical sepsis and intra-abdominal hypertension developed with the need of high ventilator pressures, decreased urine output, and ascites. Acute renal dysfunction was treated with diuretics and dopamine. With aggressive conservative management and drainage of ascites an abdominal compartment syndrome was prevented. The sepsis was caused by the bacterial species *Leclercia adecarboxylata* , which grew in the blood culture as well as in the culture of the tracheal secretion. It was successfully treated with vancomycin, gentamicin, and meropenem. After extubation on postoperative day (POD) 46, an intra-abdominal seroma persisted, which was drained on POD 60. The drain could be removed after 12 days. No recurrence of the seroma developed. The infant was discharged at the age of 3.5 months on full oral feeds. The xenograft was completely covered with skin at the age of 14 months ( Fig. 1D ). Due to a minor incisional hernia at the edge of the xenograft, we performed an incisional herniotomy at the age of 2 years. Last follow-up was at the age of 2.5 years, which showed a satisfactory outcome with good weight gain.

## Case 2

**Fig. 2:**
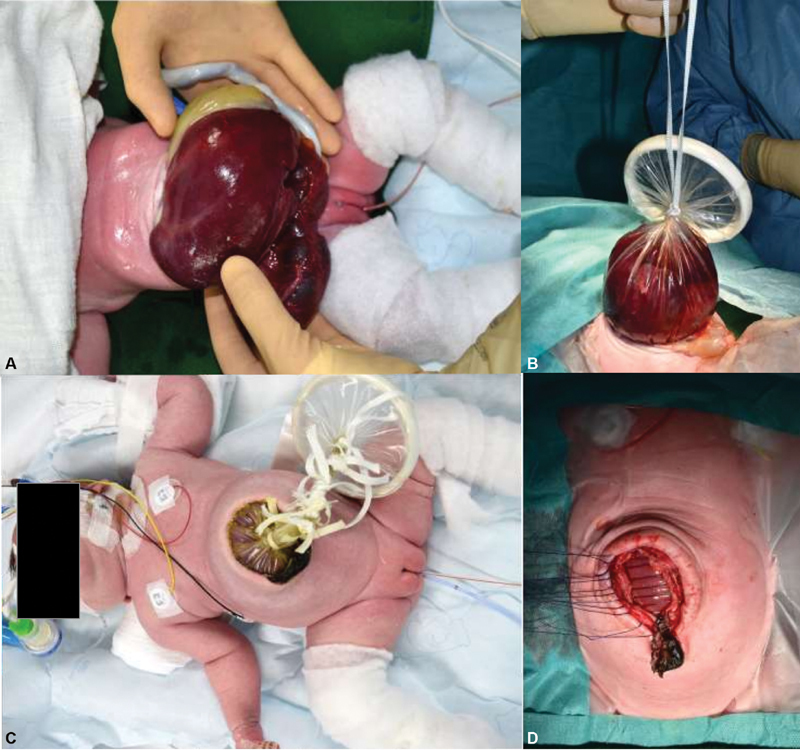
Ruptured giant omphalocele ( **A** ) after delivery, ( **B** ) application of a wound retractor, ( **C** ) after staged reduction, and ( **D** ) secondary closure of the abdominal fascia on day of life 12.

A female infant (gestational age of 38 weeks and birthweight of 3,800 g) was born via C-section with a GO. The omphalocele sac ruptured during surgical delivery, which was challenging due to severe obesity of the mother ( Fig. 2A ). Postnatally and under general anesthesia, a wound retractor (Alexis wound retractor, size S, Applied Medical) was placed under the fascia as a silo for staged reduction ( Fig. 2B ). During the following days, it was possible to slowly reduce the liver into the abdominal cavity ( Fig. 2C ). On DOL 12 secondary closure of the abdominal wall including the fascia was performed ( Fig. 2D ). The patient developed excessive pulmonary hypertension, followed by pulmonary embolism and hypertrophic cardiomyopathy of unknown origin (a syndromic cause is suspected). Enteral feeding was not tolerated. Due to severe gastroesophageal reflux disease, we performed an open Nissen fundoplication and gastrostomy at the age of 4 months. Thereafter, slow but sufficient enteral feeding was possible. The patient was discharged at the age of 6 months. After follow-up of 34 months, the child is thriving, partially fed via gastrostomy with no signs of gastroesophageal reflux or incisional hernia. However, oral feeding is still difficult with insufficient weight gain at the 7th percentile and height at the 0.1 percentile. Therefore, the child is mainly fed via the gastrostomy.

## Discussion

Despite the advancement in neonatal intensive care, the treatment of ruptured GO and giant gastroschisis is challenging and associated with high mortality. We report two patients in whom we successfully used a new technique for staged reduction, achieved abdominal closure, and long-term survival.

There are very few reports of patients with gastroschisis and total liver herniation. McClellan et al reported on liver herniation in seven of 117 patients with gastroschisis. Four of them had major liver herniation. 7 All four patients were managed with silo placement. Two of these neonates died before secondary closure of the abdominal wall, and the other two required a biologic patch for abdominal wall closure. Finally, all four patients died due to pulmonary hypoplasia between DOL 6 and 152. In the three survivors only an edge of the liver was herniated. Svetanoff et al reported on two cases of gastroschisis and total liver herniation. 9 Both children were treated with silo placement and secondary abdominal wall closure using either an acellular dermal allograft or a Stattice mesh and a negative pressure dressing. In both cases, two sheets of silastic mesh were sewn to the abdominal fascia with polypropylene and then attached to a traction/pulley system. Unfortunately, one child died on DOL 38 due to multiorgan failure. The other patient underwent 37 procedures, spent 241 days on ICU, could be discharged to a long-term care facility at 9 months, and discharged home on a ventilator at 16 months. He transitioned from conventional ventilation to high-frequency jet ventilation and then to a tracheostomy with mechanical ventilation. To our knowledge, this patient remains the only surviving patient so far with this complex congenital malformation.

The best treatment for ruptured GO is still controversially discussed in literature: staged surgical repair or delayed repair. 5 10

In our case of ruptured GO, we decided to do a staged surgical repair using an Alexis wound retractor (Alexis wound retractor, size S, Applied Medical) as a silo with secondary fascial abdominal wall closure after reduction of the abdominal organs. To the best of our knowledge, this technique of using a wound retractor for reducing a ruptured GO has not been described as a method for staged reduction in literature before.

The staged surgical repair was first described by Gross in 1948. 11 Schuster introduced a polytetrafluoroethylene reinforced sac in 1967. 12 He fixed this sac to the fascial margins with sutures and suspended the sac over the patient, letting gravity reduce the herniated viscera. Allen and Wrenn used a dacron coated silastic bag in 1969, which was sutured to the full thickness of the abdominal wall. 13 This method gained popularity in the 1980s and experienced minor modifications. Risby et al used Gore Dualmesh attached to the fascia like a silo for staged repair in gastroschisis and omphalocele. If secondary fascial closure was not possible, the mesh was left in place allowing fibrous tissue to build up beneath it or covered with skin grafts. 14 Tissue expanders have also been placed inside the abdominal cavity or in the subcutaneous or intermuscular layer to increase abdominal capacity. 15 16

Reports from developing countries describe reapproximation of the ruptured herniation sac. 17 Hereafter, delayed repair is performed with initial conservative management applying topical escharotic agents for spontaneous epithelialization (“paint and wait”). Surgical ventral hernia repair is done later when the child thrives. Aldridge et al described negative pressure wound therapy as a treatment option for ruptured omphaloceles. 18 Every reported method has associated complications, sepsis being the most frequent complication. 8 All of the staged surgical techniques above are associated with damage of the fascial edges, delay in enteral feeding, and occurrence of infection, especially after day 7. 4

In both of our cases, we repositioned the herniated viscera into the wound retractor and placed the ring of the retractor under the abdominal fascia. No sutures were needed to keep the retractor in place, thereby preserving intact fascial edges. With suspension and gentle sequential ligation of the sac from above the herniated viscera were gently reduced into the abdominal cavity over several days. Only mild ventilator pressures were needed during silo treatment. Secondary abdominal wall closure was possible in case 1 on DOL 10 and in case 2 on DOL 12.

The disadvantages of this method are infection and loss of fluid. Further obstacles of this technique include the risk of dislodgement of the wound retractor if too much force for reduction is used or not enough sedation is applied. However, in our two children we did not experience dislodgement of the wound retractor. As described by Svetanoff et al in case 1 g, our patient also needed prolonged respiratory support after secondary abdominal wall closure due to pulmonary hypoplasia. However, extubation was possible on POD 46 without the need of tracheostomy or home ventilation.

A serious problem following reduction of the intestines in the hypoplastic abdominal cavity is the development of an abdominal compartment syndrome. This complication is described as frequent as 0.6 to 10% in the pediatric surgery population. Tight abdominal wall closure of congenital abdominal wall defect is the major cause. 19 High intra-abdominal pressure can also contribute to respiratory failure and the need for ventilator support as well as renal failure. 9 Also in our case 1, intra-abdominal hypertension developed after secondary abdominal wall closure, which was successfully managed conservatively. If medical treatment fails to decrease intra-abdominal hypertension and to improve organ function, surgical decompression is needed. 19

Another complication in case 1 was the development of a postsurgical seroma after placement of the xenograft. This is described by Naji et al after using Surgisis for abdominal wall reconstruction in omphaloceles. 20

In case 1, sepsis was caused by a rare bacterial species named *L. adecarboxylata* . This species has not been described in this context before. One possibility is that these bacteria originated from the bovine patch. However, there is no proof of this hypothesis, as these bacteria were described mainly in immunocompromised patients or acquired via wounds and/or contact with aquatic environment. Lately, a few cases in immunocompetent pediatric cases with cellulitis were reported.

With the advancement in critical care medicine, it is possible to manage even giant and ruptured abdominal wall defects. Surgical intervention is part of this treatment with the aim to reduce the number of interventions, complication rate, and morbidity. The Alexis wound retractor may be a good option as it can be applied quickly without the need of sutures.

## Conclusion

Complex abdominal wall defects require an individualized treatment strategy, which cannot be standardized. Our two cases show that the wound retractor would be a good addition to the toolbox of a pediatric surgeon to treat these children. Using this device, the liver and intestines can be reduced into the abdomen with delayed closure of the abdominal wall within the first 1 to 2 weeks of life.

## Notes

New Insights and the Importance for the Pediatric Surgeon Complex abdominal wall defects require an individualized treatment strategy, which cannot be standardized. The wound retractor would be a good addition to the toolbox of a pediatric surgeon to treat these children as it is quick to apply and does not need sutures.
